# Tooth vitality preservation following odontogenic keratocyst enucleation: Systematic review and clinical implications

**DOI:** 10.4317/medoral.27666

**Published:** 2026-04-19

**Authors:** Margalida Santmartí-Oliver, Ana Federica Stran-Lo-Giudice, Francisco González Fernández-Tresguerres, Carlos Manuel Cobo-Vázquez, Cristina Madrigal Martínez-Pereda, Juan López-Quiles Martínez

**Affiliations:** 1 Department of Dental Clinical Specialties, Faculty of Dentistry, Complutense University of Madrid, Spain

## Abstract

**Background:**

Odontogenic keratocysts are locally aggressive jaw lesions with a high recurrence rate, often involving the roots of adjacent teeth. The management of these teeth remains controversial, with no consensus on the necessity of prophylactic endodontic treatment prior to enucleation. Therefore, the purpose of this study was to assess the tooth vitality response of teeth involved in the cystic lumen of odontogenic keratocysts after enucleation and evaluate the suitability of prophylactic root canal therapy.

**Material and Methods:**

A comprehensive search was conducted in PubMed, Scopus, and Web of Science following PRISMA guidelines, along with a manual search for relevant clinical articles published up to January 2025. Inclusion criteria required explicit postoperative vitality assessment of teeth involved in odontogenic keratocysts treated by enucleation, with a follow-up period of at least 6 months.

**Results:**

Four studies met inclusion criteria, analyzing 19 teeth in 5 patients, with a mean follow-up of 50 months (range: 10-84). Overall, 87.5% of teeth retained positive pulp vitality after surgery. Vitality was assessed using cold and/or electric pulp testing. All studies used 3D imaging for lesion evaluation. Longer follow-up periods were significantly associated with reduced vitality retention.

**Conclusions:**

Despite limited data, preserving pulp vitality in teeth adjacent to odontogenic keratocysts appears both viable and clinically beneficial. This conservative approach may help avoid unnecessary endodontic procedures, especially in the absence of definitive signs of necrosis. However, long-term follow-up remains essential to monitor vitality changes over time. Further high-quality studies are necessary to establish predictive success factors and standardized clinical protocols.

## Introduction

Odontogenic keratocysts (OKCs), defined as developmental jaw cysts originating from the dental lamina, have a complex history of classification due to its unique clinical and histopathological characteristics (1). Their locally aggressive behavior, high recurrence rate, and association with PTCH1 mutations and Gorlin-Goltz syndrome once led to their consideration as a tumor. However, evidence showing PTCH1 mutations in non-neoplastic cysts and the resolution of lesions after marsupialization led the WHO to reclassify OKC as a cyst in 2017, abandoning the neoplastic designation (2). This decision was reinforced in the latest edition of the WHO classification (5th edition, 2022), where the OKC continues to be categorized as a cyst lined by parakeratinized stratified squamous epithelium with hyperchromatic palisaded basal cells (3).

OKCs account for approximately 4% to 16.5% of mandibular cysts and most commonly affect individuals between the ages of 30 and 40, with a slight male predominance and a particular tendency to occur in the mandible, especially in the region of the third molar and mandibular ramus (approximately 65-80% of cases). (4) Its recurrence is around 30% and is associated with factors such as cyst size, cortical perforation, multilocular lesions, soft-tissue extension and presence of satellite cysts (5).

Clinically, OKCs are often asymptomatic when small and are diagnosed during routine radiographic examinations. However, as they grow, they can cause pain, swelling, and even facial deformities due to their expansion, which can perforate bone, affect soft tissues, displace teeth, or cause paresthesias (4 , 6).

Radiographically, OKC typically appears as a well-defined radiolucent lesion, most often unilocular but occasionally multilocular, frequently accompanied by a peripheral radiopaque border. These radiographic features are nonspecific and may resemble those of other odontogenic cysts. Definitive diagnosis is confirmed through histopathological analysis, as OKCs can be easily mistaken for radicular, residual, or dentigerous cysts, and in the case of large mandibular lesions, even ameloblastomas (1 , 4).

Regarding treatment, both conservative and aggressive options are available, depending on the cyst's size, location, and risk of recurrence. While resection was once the standard approach, current strategies increasingly favor more conservative techniques, such as marsupialization, decompression, and enucleation. In cases of cystectomy, devitalization of the involved teeth is often considered unavoidable when enucleating the cystic membrane, leading to pulp necrosis. However, there is no clear consensus on the necessity of prophylactic endodontic treatment in these cases (7). Therefore, this study aims to assess the tooth vitality response of teeth involved in the cystic lumen of OKCs after enucleation and evaluate the suitability of prophylactic root canal therapy.

## Material and Methods

Research question

This study was conducted following the Preferred Reporting Items for Systematic Reviews and Meta-Analyses (PRISMA) guidelines (8). Neither the review nor the protocol were registered in any database.

The review's focused (PIO) question was: In patients with OKCs involving the apex of adjacent teeth, is it possible to preserve pulp vitality after enucleation/cystectomy treatment? Whereby Population (P) is defined as adult patients with OKCs involving the apex of adjacent teeth; Intervention (I) as enucleation/cystectomy treatment without prophylactic root canal therapy; and Outcomes (O) as pulp vitality.

Eligibility criteria

Inclusion criteria

- Clinical studies performed in human with the presence of a OKC involving the apex of permanent vital teeth, treated by enucleation or cystectomy surgery, in which the vitality status of the involved teeth is specified.

- Randomized clinical trials (RCTs), prospective clinical trials (CCTs), observational studies (retrospective and prospective studies), case series, and case reports.

- Follow-up of at least 6 months after surgical treatment, where the vitality of the involved teeth is specified.

Exclusion criteria

- Pre-clinical studies (i.e. cadaver, animal or in vitro studies).

- Review articles.

- OKCs treated only by methods other than enucleation or cystectomy (e.g. decompression, marsupialization, or resection).

Search strategy

An electronic systematic search was conducted in PubMed (MEDLINE), Scopus (Elsevier), The Cochrane Library (Wiley), and Web of Science (Clarivate Analytics) up to January 16th, 2025, to identify all relevant studies, without restrictions on data or language. The search was performed using a combination of MeSH terms and text words, applying the filter "Humans": ("Root Canal Therapy"[Mesh] OR endodon* OR "tooth vitality" OR vital*) AND ("Odontogenic Cysts"[Mesh] OR keratocyst*) AND ("Cystectomy"[Mesh] OR "Surgical Procedures, Operative"[Mesh] OR enucleation). This strategy was adapted for each database (Supplementary Table 1. http://www.medicina.oral.com/carpeta/suppl1_27666).

The search was completed by manual screening of the references cited in selected articles and similar reviews.

Study selection

Two independent reviewers (MSO and FGFT) performed a systematic screening of studies in accordance with the predefined eligibility criteria. Following the removal of duplicates (using Mendeley Desktop, version 1.19.8, Elsevier), titles were initially assessed to exclude irrelevant publications, and abstracts were subsequently reviewed. In cases where titles or abstracts were unclear, full-text articles were obtained for further evaluation. Any disagreements during the screening process were resolved through discussion with a third reviewer (JLQ). Reasons for exclusion at each stage were documented. Inter-reviewer agreement was assessed using the Cohen kappa coefficient ().

Data extraction

Qualitative data reported in the selected studies were described using data extraction tables. Whenever possible, the following information was retrieved and entered in descriptive tables: Author(s), year of publication, country, study design, follow-up, age, gender, medical history, OKC location, assessment method of adjacent tooth apex involvement, surgical treatment, adjuvant therapies, assessment method of tooth vitality, tooth vitality evolution and recurrence.

If a case series study included other types of cysts, only data related to OKC cases were taken into account and considered as case reports.

Quality assessment

The quality of the reviewed studies was independently assessed by two reviewers (MSO and FGFT). The Joanna Briggs Institute (JBI) Critical Appraisal Checklist (9) was applied to case series and case reports, comprising ten or eight questions designed to assess both the methodological quality of the study and the clarity in reporting its results.

## Results

Study selection

**Figure 1:**
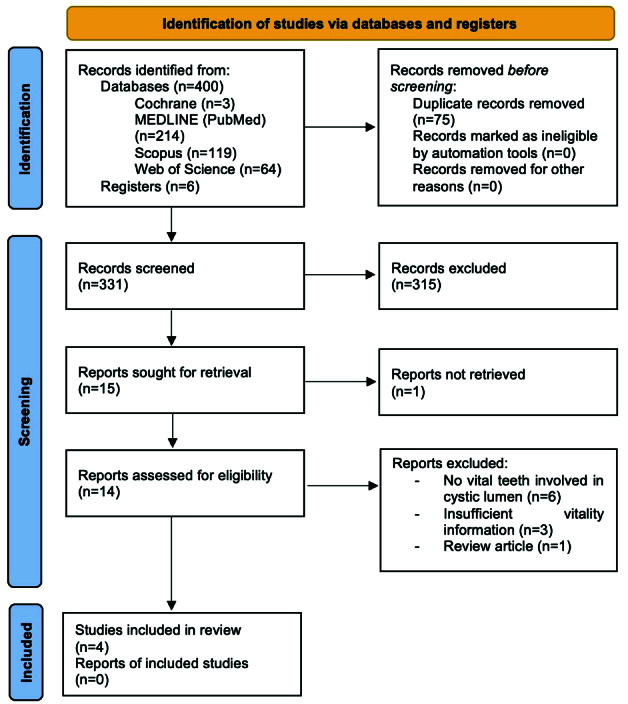
PRISMA flow diagram of the screening and selection process.

The initial electronic database search identified 400 references, with 6 additional articles found through manual searching. After removing duplicates and screening based on titles and abstracts, 15 articles were selected for full-text evaluation. However, one of these articles could not be retrieved in full text. Following the application of inclusion and exclusion criteria, 4 studies were included for data extraction and analysis. The study selection process is presented in Figure 1 and detailed information on excluded articles can be found in Supplementary Table 2 (http://www.medicina.oral.com/carpeta/suppl2_27666).

1

The calculated kappa index was 0.93, indicating a substantial level of agreement among reviewers.

Study characteristics and quality assessment

A total of 4 articles (10 - 13) were included in the present systematic review, encompassing data from 5 case reports, all published between 2014 and 2023. Table 1 describes the main characteristics of the included studies. The quality analysis of the case reports, based on the Clinical Appraisal Checklist from JBI, is provided in Table 2.

Table 1Table 2Synthesis of results

The cases involved two males (40%) and three females (60%), aged between 14 and 49 years, with a mean age of 27.6 years. The follow-up periods ranged from 10 to 84 months, with a mean duration of 50 months.

Four OKCs (80%) were located in the mandible, while one (20%) was in the maxilla.

All but one OKC were associated with one, two, or three vital teeth with their apices within the cystic lumen, whereas a single case involved ten teeth. Therefore, the total number of teeth included in this review is 19. However, two wisdom teeth were excluded because they were extracted during cyst enucleation, and one tooth was considered a dropout due to missing vitality data in the follow-up, resulting in a total of 16 teeth included.

All studies evaluated the OKC lesion using three-dimensional imaging techniques (CBCT or CT).

The final treatment in all cases was enucleation of the OKC; however, De Molon et al. (11) and Pittl et al. (13) performed a prior incisional biopsy. Additionally De Molon et al. (11) carried out marsupialization followed by lesion decompression for three months before enucleation, at which point the submerged 3.8 tooth was also extracted. Lacarbonara et al. (12) drained the cystic content before surgery and performed a Caldwell-Luc procedure on the affected sinus simultaneously with the enucleation of the OKC and extraction of the displaced ectopic third molar. Only Pittl et al. (13) used Carnoy's solution as an adjuvant treatment following enucleation.

Regarding vitality assessment, De Molon et al. (11) and Lacarbonara et al. (12) reported the vitality status of the involved teeth but did not specified the evaluation method employed. In contrast, Alharbi et al. (10) assessed vitality using both cold and Electric Pulp Test (EPT), while Pittl et al. (13) relied solely on cold testing. Fourteen of the sixteen (87.5%) monitored teeth maintained positive vitality after surgical enucleation, while two progressed to necrosis.

De Molon et al. (11) and Lacarbonara et al. (12) explicitly reported no recurrence following their respective follow-up periods of 60 and 24 months. In contrast, Pittl et al. (13) documented a recurrence one year after the initial surgery, which was managed using the same surgical approach. Alharbi et al. (10) did not provide information on recurrence.

## Discussion

This systematic review aimed to comprehensively summarize the available data on preserving the vitality of permanent teeth affected by OKC lesions and treated with enucleation or cystectomy. The results showed that 87.5% of the studied teeth retained positive vitality after surgery, with a mean follow-up of 50 months. Despite the limited number of studies specifically addressing this topic, these findings suggest that maintaining pulp vitality is achievable in most cases.

However, the management of teeth associated with cystic lesions of the jaws remains a widely debated topic, with no clear consensus on whether extraction or root canal therapy should be performed prior to or after surgery, particularly in cases of OKC. These cysts are known for their higher recurrence rates and more locally aggressive behavior compared to other odontogenic cysts. Some studies suggest that recurrent OKCs may be linked to the incomplete removal of epithelial tissue surrounding tooth roots that extend into the cyst cavity, leading to recommendations for extraction or apicoectomy to ensure complete cyst removal (7).

Various imaging modalities such as CBCT, CT, MRI, and ultrasound play a key role in evaluating cystic jaw lesions (7). Among them, CBCT stands out for its high accuracy, lower radiation exposure, and detailed 3D visualization of bone changes and lesion proximity to vital structures. Notably, all studies included in this review assessed OKC lesions using three-dimensional imaging (CBCT or CT), which is essential for accurately determining the degree of dental involvement. Two-dimensional radiography, while more accessible, lacks the spatial resolution required to evaluate critical aspects such as cortical perforation, proximity to neurovascular bundles, or the extent of root exposure-factors vital in treatment planning and prognosis.

Regarding vitality assessment, neither De Molon et al. (11) nor Lacarbonara et al. (12) specified the evaluation method. In contrast, Alharbi et al. (10) assessed vitality using both cold and electric pulp testing, while Pittl et al. (13) relied solely on cold testing. The predominance of thermal and electric pulp testing in these studies aligns with their widespread use in clinical practice due to their accessibility and ease of application. These methods are simple and efficient but do not assess true pulp blood flow. Consequently, false-negative results may occur, particularly in cases of transient pulp ischemia or neuropraxia (14 , 15). While EPT is effective in detecting healthy pulp, it has limitations when diagnosing diseased tissues due to patient variability and external factors (14 , 16). Combining EPT with Thermal Tooth Test (TPT) improves diagnostic accuracy, as TPT has higher sensitivity for detecting non-vital teeth (15 , 17). More advanced techniques, such as laser Doppler flowmetry (LDF) and pulse oximetry (PO), provide a direct assessment of pulp circulation, leading to a more precise diagnosis. However, these methods are not commonly used due to their technical demands (14 , 16). As a result, conventional tests like TPT and EPT remain the primary clinical standards. Their reliability and widespread availability make them the preferred choice in routine practice. Nonetheless, further research is needed to enhance diagnostic accuracy in cystic lesions.

Cystic expansion can lead to temporary reductions in pulp sensibility or even necrosis due to mechanical compression or ischemic effects on the inferior alveolar nerve and/or apical neurovascular bundle (7 , 15 , 18). In the initial search of our systematic review, many studies were excluded because they did not involve vital teeth associated with OKC. However, the absence of vital teeth in these studies could actually be a false negative due to the reduced response to sensibility testing rather than a true absence of vitality, that it is often preserved due to a neurovascular supply from the epithelial lining (7). Moreover, studies have suggested that pulp sensibility can recover following the removal of mechanical compression on the inferior alveolar nerve, which may induce temporary neuropraxia (15 , 16). This approach aligns with previous studies that examined pulp vitality recovery after LeFort I surgery, where vascular and nervous trauma-though less severe than in cystectomy-was also present (19 , 20). Furthermore, it has been suggested that pulp vitality testing can serve as an indicator of inferior alveolar nerve function, as traditional sensory tests often fail to detect subtle functional impairments (15 , 21). Given the complexity of determining whether pulp degeneration is permanent or reversible, further research is needed before prophylactic root canal therapy or apicoectomy becomes routine practice based solely on pulp sensibility tests (14). While nonvital teeth should receive root canal treatment, vital teeth affected by large cystic lesions may retain their viability despite significant bone loss, suggesting that temporary losses of pulp response may not always indicate irreversible damage (7). Therefore, this also supports the decision to postpone endodontic treatment until definitive pulp necrosis is confirmed.

Preserving pulp vitality in teeth affected by cystic lesions is crucial not only for maintaining tooth function but also for facilitating tissue repair and regeneration. Both Elhakim et al. (18) and Kim and Min (22) emphasize the importance of avoiding unnecessary devitalization, as vital pulp not only protects the tooth from further damage but also acts as a source of stem cells for tissue regeneration. Devitalized teeth are more susceptible to fractures and loss, which makes preserving pulp vitality particularly important in cases where cysts involve the apices of adjacent teeth (7). Maintaining pulp vitality also reduces the need for more invasive procedures and helps to minimize long-term treatment costs, which is beneficial for the patient (14). Overall, every effort should be made to preserve pulp vitality, as it significantly impacts the tooth's long-term function and prognosis.

The mechanisms involved in nerve regeneration and pulp revascularization after cyst enucleation remain subjects of ongoing research. Kim and Min (22) discusses the role of blood clot formation in the bony cavity post-enucleation, which promotes both revascularization and nerve regeneration due to its growth factor content. Additionally, dental pulp stem cells (DPSCs) from remaining vital pulp tissue may aid in reinnervation, and collateral reinnervation from nearby nerves has also been suggested. Alharbi et al. (10) further emphasizes that the restoration of blood flow after surgical osteotomy supports dental pulp regeneration. While interrupted blood circulation can cause pulp degeneration, this damage is often reversible and does not always result in devitalization. Factors such as age have been shown to affect regenerative potential, though no clear link was found between age and the incidence of necrosis, suggesting that other factors may play a more significant role, such as apical diameters, cyst type or the use of bone grafting. Recent literature has increasingly questioned the routine use of bone grafts and membrane barriers following cyst enucleation. Some studies suggest that these interventions may compromise the integrity of neurovascular bundles associated with adjacent vital teeth, thereby limiting their regenerative capacity (18 , 22). Kim and Min (22) proposes that blood clots may serve as a more biologically favorable alternative to bone grafts, providing a native scaffold enriched with growth factors that supports both vascular and neural healing. Furthermore, the application of membrane barriers in the management of large lesions has not demonstrated consistent long-term clinical benefits. Notably, the introduction of radiopaque bone substitutes within the enucleated cavity may also complicate radiographic follow-up by obscuring or mimicking signs of lesion recurrence, thus posing diagnostic challenges in postoperative monitoring.

Although this review focused on odontogenic keratocysts, evidence from other cystic lesions also supports the potential to preserve pulp vitality following enucleation. For instance, Niu et al. (14) implemented surgical modifications that successfully maintained pulp vitality in 84 of 103 involved teeth across cases of radicular, dentigerous, and nasopalatine cysts; these surgical modifications involved removing bone 5mm above the root apex to safeguard the blood supply, incising only the epithelial lining of the capsule wall and part of the fibrous lining, thereby partially preserving the fibrous tissue. This approach may help maintain papillary, pulpal, and periodontal stem cells, and covering the root apex with blood clots during surgery further facilitates the preservation and restoration of tooth vitality. Similarly, Abu-Id et al. (23) reported preservation of sensibility in all mandibular teeth involved with a large glandular odontogenic cyst one year after cystectomy with Carnoy's solution. Asgary and Parhizkar (24) described a case where correct diagnosis and protection of adjacent teeth enabled vitality preservation despite a large periapical lesion. Kim and Min (22) documented full recovery of pulp sensibility in lower incisors exposed during surgical removal of a periradicular lesion. Nabil et al. (25) emphasized the importance of long-term follow-up to assess tooth vitality, reporting cases of pulp responsiveness recovery even up to 300 days after surgery in radicular and dentigerous cysts, concluding that tooth vitality should be re-evaluated for at least ten months postoperatively before considering root canal treatment. These findings reinforce the notion that with careful surgical planning and pulp-protective strategies, vitality can be preserved even in teeth adjacent to extensive cystic pathology. Given that odontogenic keratocysts fall under the same category of odontogenic cysts in the current WHO classification (5th edition, 2022) (3), they should not necessarily be managed differently, despite their higher recurrence rates.

Alternatively, in cases where teeth are involved in large cystic lesions, non-invasive techniques like marsupialization and decompression can be also useful to preserve vitality. Marsupialization followed by enucleation or curettage has demonstrated success in reducing the size of large cystic lesions such as OKCs, with low recurrence rates and minimal functional and aesthetic damage (11). This approach helps preserve vital anatomical structures and allows for the gradual reduction of cyst size before enucleation, which minimizes the risk of damage to adjacent teeth and other vital structures (26). While these techniques have proven effective, they require patient cooperation and extended follow-up, making them less suitable for all patients (18). Nevertheless, marsupialization and decompression serve as valuable initial steps in the treatment of large lesions and can significantly reduce the need for more invasive treatments.

Alharbi et al. (10) investigated the incidence of pulp necrosis in vital teeth following the surgical removal of adjacent jaw lesions, reporting necrosis in 54.6% of cases and vitality preservation in 45.4%. While no significant associations were found with demographic or lesion-related variables, follow-up duration was the only factor significantly influencing vitality outcomes (p=0.040), with higher vitality retention observed in teeth monitored for shorter periods (p=0.01). These findings highlight the progressive risk of vitality loss over time and underscore the need for long-term follow-up. Given the high recurrence rate of odontogenic keratocysts and the potential for delayed changes in pulp status, extended monitoring is essential. In our review, the mean follow-up was 50 months, with a range of 10 to 84 months, supporting a recommendation of a long-term monitoring, preferably lifelong.

This systematic review highlights the potential for preserving pulp vitality in teeth associated with OKCs, even in extensive lesions. Although prophylactic root canal therapy has traditionally been used to prevent complications like apical periodontitis or superinfections, current evidence favors a more conservative and individualized approach. Clinical decisions should be guided by factors such as lesion size, proximity to apices, and imaging findings, rather than routine devitalization. The capacity for pulp recovery after decompression or enucleation, along with advances in diagnostics and regenerative biology, supports avoiding unnecessary devitalization. As shown in our findings and in long-term studies like Alharbi et al. (10), pulp vitality can be maintained over time, emphasizing the importance of extended follow-up. Ultimately, treatment should aim to preserve vitality whenever possible, balancing surgical success with long-term function and patient quality of life.

However, this review has limitations, notably the small number of included studies, limited sample sizes, and variability in surgical protocols, vitality assessment methods, and follow-up periods. Further research is needed to validate these findings and clarify the factors influencing outcomes.

## Conclusions

Within the limitations of this systematic review, it can be concluded that preserving the vitality of teeth involved in or adjacent to jaw cystic lesions is a viable and potentially advantageous option, especially in the absence of definitive clinical or radiographic signs of pulp necrosis. Emphasizing the importance of regular follow-up is essential, as repeated evaluations over time may reveal changes in sensitivity test responses. This conservative approach can help clinicians make more informed decisions and potentially avoid unnecessary endodontic treatment. However, due to the heterogeneity and limited number of high-quality studies currently available, further studies are required to establish a more definitive conclusion.

## Figures and Tables

**Table 1:** Main characteristics of the included studies. NR: Not reported. NS: Not significant. M: Male. F: Female. EPT: Electric pulp test. CBCT: Cone-beam computed tomography. CT: Computed tomography. CS: Carnoy’s solution.

Authors, year	Country		Study design (subjects)		Follow-up (months)	Gender & Age (years)	Medical history			OKC location		
Alharbi et al., 2023 [10]	Saudi Arabia		Cases Reports (2)		1072	M - 18F - 49	NRNR			Posterior mandiblePosterior mandible		
De Molon et al., 2015 [11]	Brazil		Case Report		60	M - 15	No systemic diseases or medication			Posterior mandible		
Lacarbonara et al., 2014 [12]	Italy		Case Report		24	F - 14	NR			Maxillary sinus		
Pittl et al., 2017 [13]	Austria		Case Report		84	F - 42	NS			Anterior & posterior mandible		
Authors, year		Apex involvement assessment method		Surgical treatment				Tooth vitality assessment method	Tooth vitality evolution		Recurrence	
Alharbi et al., 2023 [10]		CBCT		Enucleation				Cold test & EPT	3.5: Vital4.4 & 4.5: Necrosis		NR	
De Molon et al., 2015 [11]		OPG and CT		Incisional biopsy + marsupialization & 3-months decompression + enucleation & extraction 3.8				NR	3.6: NR3.7: Vital3.8: Extraction		No	
Lacarbonara et al., 2014 [12]		OPG + CT		Drainage + antral-cystectomy (Caldwell-Luc technique) / enucleation + extraction 2.8 + interocclusal splinting with wrought metal wire				NR	2.6 & 2.7: Vital2.8: Extraction		No	
Pittl et al., 2017 [13]		OPG + CT		Biopsy + enucleation + CS + plate				Cold test	3.5 to 4.5: Vital		Yes (1 year)	

NR: Not reported. NS: Not significant. M: Male. F: Female. EPT: Electric pulp test. CBCT: Cone-beam computed tomography. CT: Computed tomography. CS: Carnoy’s solution.

**Table 2:** Evaluation of the quality of Case Reports using the JBI Critical Appraisal Tool (Munn et al., 2020).

Study	Alharbi et al. [10]	De Molon et al. [11]	Lacarbonara et al. [12]	Pittl et al.[13]
1. Were patient's demographic characteristics clearly described?	-	+	-	+
2. Was the patient's history clearly described and presented as a timeline?	+	+	+	+
3. Was the current clinical condition of the patient on presentation clearly described?	+	+	+	+
4. Were diagnostic tests or assessment methods and the results clearly described?	+	+	+	+
5. Was the intervention(s) or treatment procedure(s) clearly described?	+	+	+	+
6. Was the post-intervention clinical condition clearly described?	-	+	+	+
7. Were adverse events (harms) or unanticipated events identified and described?	+	+	+	+
8. Does the case report provide takeaway lessons?	+	+	+	+
Global evaluation	Include	Include	Include	Include

+: Yes. -: No. ?: Unclear. NA: Not Applicable.

## Data Availability

Declared none.
